# Is Rumination after Bereavement Linked with Loss Avoidance? Evidence from Eye-Tracking

**DOI:** 10.1371/journal.pone.0104980

**Published:** 2014-08-20

**Authors:** Maarten C. Eisma, Henk A. W. Schut, Margaret S. Stroebe, Jan van den Bout, Wolfgang Stroebe, Paul A. Boelen

**Affiliations:** 1 Utrecht University, Department of Clinical and Health Psychology, Utrecht, Netherlands; 2 University of Groningen, Department of Experimental Psychopathology and Clinical Psychology, Groningen, Netherlands; 3 Utrecht University, Department of Social and Organizational Psychology, Utrecht, Netherlands; 4 University of Groningen, Department of Social Psychology, Groningen, Netherlands; University of Florida, United States of America

## Abstract

Rumination is a risk factor in adjustment to bereavement. It is associated with and predicts psychopathology after loss. Yet, the function of rumination in bereavement remains unclear. In the past, researchers often assumed rumination to be a maladaptive confrontation process. However, based on cognitive avoidance theories of worry in generalised anxiety disorder (GAD) and rumination after post-traumatic stress disorder (PTSD), others have suggested that rumination may serve to avoid painful aspects of the loss, thereby contributing to complicated grief. To examine if rumination is linked with loss avoidance, an eye-tracking study was conducted with 54 bereaved individuals (27 high and 27 low ruminators). On 24 trials, participants looked for 10 seconds at a picture of the deceased and a picture of a stranger, randomly combined with negative, neutral or loss-related words. High ruminators were expected to show initial vigilance followed by subsequent disengagement for loss stimuli (i.e., picture deceased with a loss word) in the first 1500 ms. Additionally, we expected high ruminators to avoid these loss stimuli and to show attentional preference for non-loss-related negative stimuli (i.e., picture stranger with a negative word) on longer exposure durations (1500–10000 ms). Contrary to expectations, we found no evidence for an effect of rumination on vigilance and disengagement of loss stimuli in the first 1500 ms. However, in the 1500–10000 ms interval, high ruminators showed shorter gaze times for loss stimuli and longer gaze times for negative (and neutral) non-loss-related stimuli, even when controlling for depression and complicated grief symptom levels. Effects of rumination on average fixation times mirrored these findings. This suggests that rumination and loss avoidance are closely associated. A potential clinical implication is that rumination and grief complications after bereavement may be reduced through the use of exposure and acceptance-based therapeutic techniques.

## Introduction

Is rumination after bereavement linked with loss avoidance? Evidence from eye-tracking.

Ruminative thinking, broadly defined as repetitive and recurrent, self-focused thinking about negative emotions and/or negative events [1], has been identified as a risk factor in adjustment to bereavement [2], [3]. Rumination after loss both concurrently and prospectively predicts general distress and symptoms of depression, posttraumatic stress and complicated grief [2], [4]–[9]. Since levels of rumination may be reduced through therapy [10], it is crucial to understand the pathways through which rumination contributes to the development and persistence of mental health problems after loss. After all, this information could be used to increase efficacy of therapeutic interventions for bereaved individuals with high levels of rumination and complicated grief.

Despite a large body of research on causes, correlates and consequences of rumination in depression [11], it is not yet entirely clear in what way rumination contributes to mental health problems after bereavement. In the past, many researchers more or less explicitly assumed rumination after stressful events to be a confrontation process [3], [12]–[13]. For instance, Nolen-Hoeksema and colleagues, who conducted the first large-scale studies on rumination in bereavement [8], [9], considered rumination to be the “opposite form of coping” to denial/suppression, referring to this process as “the polar opposite of avoidance and denial” [3], [12]. According to their Response Styles Theory (RST), rumination has various negative effects because bereaved ruminators repeatedly *confront* themselves with their loss-related problems and emotions. As a consequence, rumination i) increases accessibility of negative thoughts, ii) interferes with problem solving, iii) impedes instrumental behavior and iv) drives away social support, thereby contributing to depression [3], [11]. Notably, Nolen-Hoeksema and colleagues adjusted their original position on rumination recently to include a link with behavioral avoidance. According to this extension of RST, rumination takes up time and increases feelings of hopelessness about the current situation, thereby contributing to inactivity and social withdrawal. However, they still explicitly rejected the idea that rumination is a cognitive avoidance process [11].

In a similar vein, self-regulation theorists proposed that rumination consists of a recurrent focus on discrepancies between a current situation and a desired goal or state and is motivated by the intention to reduce such discrepancies (e.g., [14]). Bereaved individuals may thus repeatedly focus on the loss and loss-related feelings, in order reduce discrepancies in mood state or to come to terms with the loss [13]. However, in the absence of any progress in reducing loss-related discrepancies, persistent focus on the loss-related problems will increase negative mood and depression.

In contrast to the notion that ruminators confront negative feelings and problems, other researchers have argued that rumination may be linked with or similar to avoidance, which could (at least partly) account for its maladaptive outcomes [11], [15]–[20]. In fact, scientists from many different research areas, including the field of generalised anxiety disorder [21], [22], post-traumatic stress disorder [16] and depression [23], have proposed that repetitive thinking styles such as rumination and worry may be cognitive avoidance processes. Of particular pertinence to the current investigation, Boelen and colleagues [15] (cf. [16]) suggested that bereaved individuals with complicated grief may engage in continuous rumination about their own reactions and the reasons why the loss occurred as a means to “escape” from having to admit the reality of the loss and the emotions linked with it. Stroebe and colleagues [19] similarly state in their Rumination as Avoidance Hypothesis (RAH) that rumination following bereavement may function as a “distraction” from more emotionally-laden topics, which may be too overwhelming to confront, such as the reality of the loss. Such avoidance of painful aspects of the loss consequently interferes with acceptance of the loss [19], [24], and/or integration of memories about the loss with autobiographical memories about the self and the relationship with the lost person [15] (cf. [16]), fueling the persistence of complicated grief.

Despite the potential theoretical implications of a link between rumination and loss-related avoidance, research on this topic has been limited. Nevertheless, some recent studies provided support for an association between rumination and avoidance after bereavement. First, in a cross-sectional survey among female widowed survivors of war, a moderate positive correlation was reported between the trait tendency to ruminate and experiential avoidance, defined as avoidance of internal experiences such as memories, thoughts and feelings [25]. Second, in a multiple mediation study in a sample of bereaved individuals, experiential avoidance and thought suppression longitudinally mediated the relationship between grief rumination and complicated grief symptom change [17]. These findings are in line with a larger body of survey research in non-clinical and depressed samples supporting an association between rumination, cognitive and/or experiential avoidance and psychopathology [20], [23], [25]–[29].

To our knowledge, no research to date has established a relationship between rumination and behavioral - rather than self-report - measures of avoidance in bereaved individuals. However, some researchers have attempted to explicitly investigate such a link in non-bereaved samples. For example, Giorgio and colleagues [23] invited college students high and low on trait rumination to participate in a dichotic listening task in which neutral words were presented to the non-dominant ear, whilst a depressive and a neutral story were presented in the dominant ear. Contrary to expectations, no differences were found between high and low ruminators on the number of neutral words they recognised after the task, indicating that high ruminators did not have a preference for neutral material when this was simultaneously presented with general, negative information. In a second task, high and low ruminators were induced to engage in relaxation or rumination, after which they received a depressive mood induction (i.e., imagining the death of a loved one). They expected that high ruminators in the relaxation condition would show a physiological response (i.e., increase in heart rate) to the imagination exercise, whereas high ruminators in the rumination condition would not. Interestingly, they found that high ruminators in the relaxation and rumination conditions did not differ in their physiological response to the imagination exercise. However, the expected difference was found in the low rumination group, suggesting that the emotional suppression effect of rumination is only observed in people who do not ruminate regularly. The authors hypothesized that this difference may potentially be the result of the fact that the depressive mood induction could have led high ruminators in the relaxation condition to ruminate, whereas low ruminators in the relaxation condition were less inclined to do so. These results therefore provide preliminary evidence for an avoidant function of rumination for individuals exposed to personally-relevant threatening material (i.e., imagining the death of a loved one).

In the current investigation, we aimed to extend findings on the relationship between rumination and avoidance using a different method, that is, by studying the association between rumination and attention for loss and non-loss stimuli in a recently bereaved sample. The main reason for selecting this approach was that the study of attention is a broadly accepted, face-valid measure for the analysis of avoidance and confrontation processes [30]. Since hypotheses on the avoidant function of rumination state that rumination is focused on general negative topics, yet functions to avoid the reality of the loss, we studied attention for loss-related stimuli when simultaneously presented with non-loss-related negative stimuli (see: ‘Stimuli development and presentation’ in the Methods section).

To our knowledge, there has been no previous research on rumination and attention for personally-relevant threatening material. However, a recent review supports a link between rumination and cognitive and attentional biases toward general negative material, such as negative words and sad faces [31]. Notably, some researchers have aimed to clarify the link between rumination and attention for general negative material using dot-probe tasks in non-bereaved samples [32]–[33]. In the dot-probe task, stimuli are presented in different locations on a computer screen. After the display is terminated, a neutral probe appears in the former location of one of the stimuli. Participants’ responses to the probe are timed and used to infer the allocation of attentional resources because responses will be faster to probes that appear in an attended rather than unattended area. For example, Donaldson and colleagues [30] reported that depressed individuals compared to non-clinical controls showed a preference for negative words when these were presented with neutral words, but only at the longer (1000 ms) and not at shorter (500 ms) exposure durations. This effect was more pronounced for high trait ruminators, compared to low trait ruminators, but inductions of rumination and distraction did not influence results. Similarly, others found that depressive rumination was related to attentional bias for sad faces as opposed to neutral faces in depressed individuals at 1000 ms [33]. In sum, these studies indicate that a stronger tendency to ruminate is related to attention biases toward general negative material, yet only after longer exposure durations.

Whereas hypotheses on rumination and attentional patterns for general negative material can be formulated on the basis of previous investigations, the relationship between rumination and attention for personally-relevant threatening material (i.e., death of a loved one) has not previously been investigated. Therefore, we predicted that higher levels of rumination would be associated with typical anxious attentional response patterns for stimuli that represent the loss. In a recent review, Ouimet and colleagues [30] described this fearful pattern of attention as being characterised by initial, subconscious orientation toward threatening stimuli (0–500 ms), followed by attentional disengagement (500–1500 ms) and avoidance of threatening stimuli beyond exposure times of 1500 ms (e.g., [32]–[33]). Accordingly, we expected high ruminators but not low ruminators to show initial vigilance and subsequent avoidance of stimuli that represent the loss and, as mentioned, a preference for general negative material at longer exposure durations.

In order to assess such attention patterns, we employed eye-tracking technology, as it offers a number of distinct advantages over other attentional tasks, such as the dot-probe paradigm. First, eye-tracking enables the study of patterns of attention, rather than the attention to certain stimuli at a fixed moment in time. It therefore offers a more fine-grained perspective on viewing behavior, rather than giving a mere ‘snapshot’ of attention [35]. Second, eye-tracking is a more reliable measure of attention for emotional material than dot-probe tasks, especially for longer exposure durations [36]. Finally, as eye tracking does not employ measurement of reaction times, this limits the effects of age and familiarity with computer tasks on outcomes. This may be of particular importance in the current investigation because the bereaved population is on average older than the general population.

In short, we aimed to assess the link between rumination and attentional avoidance of loss cues in a bereaved sample. Our predictions with regard to gaze times (total time spent looking at a stimulus in a specific interval) for this study were: High ruminators, compared to low ruminators, will show increased attention for stimuli that represent the loss on short exposure durations (0–500 ms). High ruminators, compared to low ruminators, will consequently disengage attention for stimuli that represent the loss during longer exposure durations (500–1500 ms). High ruminators, compared to low ruminators, will continue to divert attention away from stimuli that represent the loss on extended exposure durations (1500–10000 ms). These avoidant attention patterns were expected to be mirrored in attention for non-loss-related negative cues. That is, we expected that high ruminators, in comparison to low ruminators, would show heightened attention for non-loss-related negative stimuli on extended exposure durations (1500–10000 ms). Finally, we predicted high ruminators would show shorter average fixation times (time spent looking at a stimulus each time one looks at it) for loss stimuli and longer average fixation times for non-loss-related negative stimuli, when compared to low ruminators. All effects were expected to remain significant even after controlling for loss-related distress, operationalised as symptom levels of depression and complicated grief.

## Method

### Sample

Participants were pre-selected on the basis of their scores on a scale to measure grief-specific rumination, the Utrecht Grief Rumination Scale (UGRS: [2], [7]), from a database of recently bereaved adults who previously participated in a questionnaire study, and were asked and agreed to participate in an additional study. Only participants scoring in the lowest and highest quartile of the UGRS (Range: 15–75) in this previous study were selected for participation in the current investigation. During the present study the UGRS was re-administered to assess present levels of grief rumination. The total sample consisted of 54 participants, divided into 27 high ruminators (*M* UGRS score = 50.19, *SD* = 9.88), and 27 low ruminators (*M* UGRS score = 27.00, *SD* = 4.29). All participants had normal or adjusted to normal vision, as evidenced by their ability to read instructions on a computer screen before the start of the eye-tracking task. Sample characteristics are shown in Table 1.

**Table 1:** Sample characteristics of high and low ruminators.

	Low ruminators (N = 27)	High ruminators (N = 27)
Demographic variables		
Sex (N (Valid %))		
Male	3 (11)	5(18)
Female	24 (89)	22 (82)
Age in years (M (SD))	54.5 (8.4)	54.0 (11.9)
Loss-related variables		
Deceased is (N (Valid %)		
Partner	11 (41)	14(52)
Child	6 (22)	9 (33)
Parent	6 (22)	1 (4)
Sibling	4 (15)	3 (11)
Cause of loss (N (Valid %))		
Natural causes (e.g., illness, heart failure)	21 (78)	20 (74)
Accident	3 (11)	6 (22)
Suicide	3 (11)	1 (4)
Loss was (N (Valid %))		
Expected	11(41)	5 (19)
Unexpected	14 (52)	20 (74)
Both or neither	2 (7)	2 (7)
Time since loss in months (M (SD))	25.6 (10.2)	26.7 (10.7)
Psychological variables		
Grief rumination (M (SD))*	27.0 (4.3)	50.2 (9.9)
Symptom levels of depression (M (SD))*	10.3 (9.8)	27.1 (11.4)
Symptoms of complicated grief (M (SD))*	27.8 (20.2)	63.2 (22.0)

Note. Categories with fewer than 5 observations were excluded from. χ^2^- analyses. * = significant difference at *p*<001.

### Procedure

**Figure 1:**
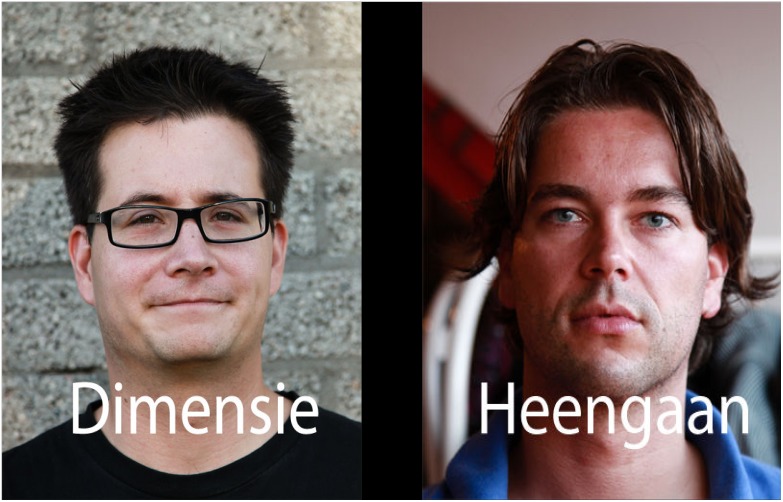
An example of stimuli presented in the eye-tracking task. Note. A translation of “Dimensie” and “Heengaan” is “Dimension” and “Passing”, respectively. In this trial the right picture-word combination is the loss-reality stimulus (deceased+loss word) and the left picture-word combination is a neutral stimulus (stranger+neutral word). The persons in this figure have consented to their pictures being published in an open access journal.

This research was conducted with the approval of the Institutional Review Board of GGZ Nederland (METIGG) and has been conducted in line with the principles expressed in the Declaration of Helsinki. Before the start of the study, each participant was informed about the study and provided written informed consent. The pictures of two people are shown in this manuscript (Figure 1). The individuals in this manuscript have given written informed consent (as outlined in PLOS consent form) to publish these case details.

The study consisted of two parts. First, each participant filled out a battery of questionnaires (see Section ‘Questionnaires’). Second, the eye tracking system was calibrated and validated and participants read the instructions for the eye tracking task on the computer screen, shown approximately 60 cm in front of them. Participants were informed that they would be looking at pictures of the deceased and pictures of a stranger combined with various words (see section ‘Stimuli development and presentation’) for 10 seconds each time, for approximately 6 minutes. Participants were told they could look at the pictures as if they were looking at a photo album, and were free to gaze at any part of the screen, but not outside the screen. In between trials, a fixation cross would be shown for five seconds in the centre of the screen and participants were asked to look at this fixation cross if nothing else was depicted on the screen. This fixation cross was used to prevent participants from looking at the left or right side of the screen before the start of the trial. After completion of two additional tasks (not reported in this paper) participants were debriefed and received 20 euros for their participation and a travel expense form.

### Questionnaires

#### Sociodemographic and loss-related variables

Demographic characteristics of the participant (age, sex and education level) and characteristics of the loss (relationship with deceased, time since the loss, cause of death and expectations about the death) were assessed with a background questionnaire.

#### Grief rumination

The 15-item Utrecht Grief Rumination Scale (UGRS) was used to measure grief-specific rumination, defined as recurrent, repetitive and self-focused thoughts about the causes and consequences of the loss and related negative feelings [2], [7]. Participants indicated how frequently they have experienced certain thoughts during the past month on a 5-point scale ranging from 1 (*never*) to 5 (*very often*). Examples of items are: “How often in the past month did you analyze if you could have prevented the loss?”, and: “How often in the past month did you try to understand your feelings about the loss precisely?” The UGRS is a reliable and valid measure of grief-related rumination [2], [7].

#### Symptoms of depression

As a first control variable we assessed depressive symptoms with the Center for Epidemiologic Studies Depression Scale, CESD Scale [37]–[38]. On the 20-item CESD Scale respondents indicated how often they had experienced certain depressive feelings or exhibited certain depressive behavior in the past week on a 4-point scale ranging from 0 (*rarely*) to 3 (*most of the time*). Multiple studies have confirmed the reliability and validity of the CESD Scale in clinical and non-clinical populations [38].

#### Symptoms of complicated grief

As a second control variable we used symptoms of complicated grief experienced in the preceding month, measured with the Inventory of Complicated Grief-Revised, ICG-R [39]–[40]. The Dutch version consists of 29 statements about the frequency and intensity of complicated grief symptoms. Answers are given on a five-point scale ranging from 0 (*almost never*) to 4 (*always*). Studies in sub-clinical samples of bereaved individuals have corroborated the reliability and validity of the ICG-R [40].

### Stimuli development and presentation

When considering stimuli development, it is important to note that rumination has been proposed to serve as a strategy to avoid the ‘reality of the loss’. Therefore, a crucial step in our research was to develop stimuli that represent this reality. Since threat-relevant verbal material generally generates weaker emotional and attentional responses than threat-relevant images [41], we decided not to rely exclusively on verbal stimuli. When considering pictorial stimuli, only pictures of the deceased person were considered both personally-relevant and relatively easy to standardise across participants. An additional advantage of such stimuli is that they can be matched with neutral pictures (i.e., pictures of a stranger). However, a potential problem with pictures is that they can generate different types of associations in different bereaved individuals. For example, some mourners may recall a fond memory when looking at a picture of the deceased, while others are reminded of the funeral. In order to ensure that participants associate pictures of the deceased with the loss, two picture types (deceased, stranger) were combined with different words, namely loss-related, negative, and neutral words (cf. [42]). The crucial stimulus, representing the loss, is a picture of the deceased combined with a loss-related word. Three other stimuli types were loss-related, but ambiguous (picture deceased+neutral word, picture deceased+negative word, picture stranger+loss-related word). Two other stimuli were non-loss-related and negative (picture stranger+negative word) and neutral (picture stranger+neutral word) in valence.

In order to create the picture-word-composites described above, a standardised procedure was used. Prior to the experiment, each participant was asked to provide a high quality picture of their deceased loved one. This picture was matched with a picture of a stranger on the basis of gender, age and picture type (i.e., portrait, standing outside, standing inside, sitting inside, sitting outside). Occasionally, pictures of the deceased were adjusted with Photoshop (e.g., by centring the deceased in the middle of the picture and/or removing distracting background characteristics) to ensure maximum comparability of both images.

Moreover, 48 different words, including 3 different word types, namely loss-related words (e.g., loss, death), negative words (e.g., down, sad) and neutral words (e.g., circle, square) were chosen for this study. Words of each type were matched on word frequency and word length. Beforehand, 5 independent judges rated each word on valence, on a 5-point scale ranging from −2 (very negative) to +2 (very positive), and on the extent to which they perceived these words to be associated with loss, on a 5-point scale ranging from 1 (not at all) to 5 (very much). Valence ratings for loss-related and negative words were more negative than neutral words, *t*(30) = −8.06, *p*<.001, and, *t*(30) = −9.14, *p*<.001, respectively. Loss-related words were considered to be more strongly associated with loss than negative, *t*(30) = −16.21, *p*<.001, and neutral words, *t*(30) = −11.03, *p*<.001.

Finally, each picture type (i.e., deceased, stranger) was combined with each word type (i.e., loss-related, negative, neutral) 8 times, forming a total of 48 picture-word composites, that is, 8 composites of 6 types (i.e., deceased-loss, deceased-negative, deceased-neutral, stranger-loss, stranger-negative, stranger-neutral). During the experiment these stimuli-composites were presented in pairs. On 24 trials of 10 seconds, a picture of the deceased and a picture of a stranger, each combined with a different word type, randomly appeared on the left or right side of the screen. Each trial contained a picture of the deceased and a picture of a stranger. Each word was used only once across all trials. All stimuli types appeared equally often on the left and right side of the screen. All stimuli were 800 pixels wide and 1100 pixels high and were separated by 200 pixels during presentation. The stimuli were presented against a black background on a 19-inch monitor with a 1680×1050 pixel resolution. For an example of possible stimuli combinations depicted on the screen see Figure 1. Eye fixations were measured at 8 ms intervals for 10 seconds of presentation time on each trial with a Tobii X120 eye tracker.

### Design and statistical analyses

The first 1500 ms of each 10 seconds of presentation time in each trial were analysed in detail, because we expected high ruminators, compared to low ruminators, would show vigilance and disengagement of loss stimuli in this interval. Therefore, the first 1500 ms were divided into 3 intervals of 500 ms each. As we expected to find different attentional patterns for high and low ruminators after 1500 ms, the last 8500 ms interval was analysed separately. For each interval, we calculated the average gaze time (i.e., average overall time spent looking at a stimulus during an interval) for each stimulus type. Since we were also interested in average fixation times (i.e., average time spent looking at a specific stimulus each time one looks at it), these were also calculated for each stimulus type over the full 10 seconds of presentation time.

As mentioned previously, three hypotheses were tested. First, we expected that high ruminators, compared to low ruminators, would show differential attention patterns for stimuli that represent the loss (i.e., picture deceased+loss word) in the first 1500 ms, showing a vigilance-avoidance pattern for such stimuli. To test this prediction, we conducted a 2×6×3 repeated measures analysis with between level factor group (high vs. low rumination) and within factors stimuli composites, consisting of 6 picture-word combinations (deceased-loss, deceased-negative, deceased-neutral, stranger-loss, stranger-negative, stranger-neutral), and time (0–500 ms, 500–1000 ms, 1000–1500 ms) on average gaze time.

Second, we expected high ruminators, compared to low ruminators, to avoid loss stimuli, in favour of non-loss-related negative stimuli for extended exposure durations (1500 ms–10000 ms). To examine this difference, a 2×6 analysis of variance with between factor group (high vs. low ruminators) and within factor stimuli composites (deceased-loss, deceased-negative, deceased-neutral, stranger-loss, stranger-negative, stranger-neutral) was conducted on average gaze time in the final 1500–10000 ms interval of presentation time.

Third, we expected that avoidance of loss stimuli and preference for non-loss-related negative stimuli shown by high ruminators, when compared to low ruminators, would also be reflected in average fixation times. To test for such group differences, we conducted a 2×6 analysis of variance with the between factor group (high vs. low ruminators) and within factor stimuli composites (deceased-loss, deceased-negative, deceased-neutral, stranger-loss, stranger-negative, stranger-neutral) and the dependent variable average fixation time in the full 10 seconds of presentation time. Finally, if these overall tests showed significant results, we conducted post-hoc test to examine differences between high and low ruminators on average gaze time and fixation time for each stimulus type.

As mentioned, the effects of symptom levels of depression and complicated grief were taken into account on all analyses by including them as covariates. Notably, there is some debate as to whether analysis of covariance can be used if covariates have high correlations with the independent group variable and the dependent variable [43]. However, analysis of covariance is essentially equivalent to a multiple regression analysis with one categorical and one or more continuous independent variables. Therefore, although analysis of covariance does not “equate” groups on pre-existing differences, it does permit estimation of direct effects of the group variable on the dependent variable, controlling for effects of continuous independent variables [44]. All analyses were conducted with the Statistical Package for Social Sciences 20.0 (SPSS 20.0).

## Results

### Preliminary analyses

#### Participant exclusion and apparatus error

Since we considered it unethical to restrict participants’ moving potential, while watching highly emotional pictures, we did not use a chin-rest during experimental tasks. As a consequence, the eye tracker failed to register gaze direction for 7 participants (4 high and 3 low ruminators) and 80% of all gaze directions for 1 participant (1 high ruminator). Gaze times for a specific stimulus type in a specific interval (i.e., 0–500 ms, 500–1000 ms, 1000–1500 ms, 1500–10000 ms) were excluded from the analyses if less than fifty percent of gaze times on all relevant trials could be determined (3.9% of all intervals). Two participants were excluded on the basis of their attention patterns. Although first fixation errors (i.e., not looking at the fixation cross when a trial started) were uncommon (*M* = 2.15, *SD* = 2.25), one participant had 18 fixation errors in 24 trials and was therefore excluded. Another participant was excluded because, relative to her group (high ruminators), the majority of her mean gaze times were outliers (i.e., larger than the overall mean+−3 *SD*s). In the main analyses, we included the data from 44 participants (22 high and 22 low ruminators).

#### Group characteristics

As shown in Table 1, no significant differences were found between high and low ruminators on gender, χ^2^ (1) = .44, *p* = .70, age, *t*(52) = 0.19, *p* = .85, time since loss, *t*(52) = −0.38, *p* = .71, expectedness of the loss, χ^2^ (2) = 3.31, *p* = .69, cause of death, χ^2^ (3) = 2.02, *p* = .36, and the relationship with the deceased, χ^2^ (3) = 4.67, *p* = .20 High ruminators, compared to low ruminators, did show elevated levels of symptom levels of depression, *t*(52) = −5.71, *p*<.001, and complicated grief, *t*(52) = −6.16, *p*<.001.

### Main analyses

#### Analyses of gaze times from 0–1500 ms

As mentioned, to investigate early attentional bias toward loss stimuli and subsequent avoidance of these stimuli in the first 1500 ms a 2×6×3 repeated-measures analysis on gaze time was executed. This analysis did not yield a significant overall effect, *F*(12,22) = 1.21, *p* = .34, *p*η^2^ = .40. The presence of a vigilance and avoidance pattern of attention for loss stimuli for high ruminators in comparison to low ruminators could therefore not be confirmed.

#### Analyses of gaze times from 1500–10000 ms

**Figure 2:**
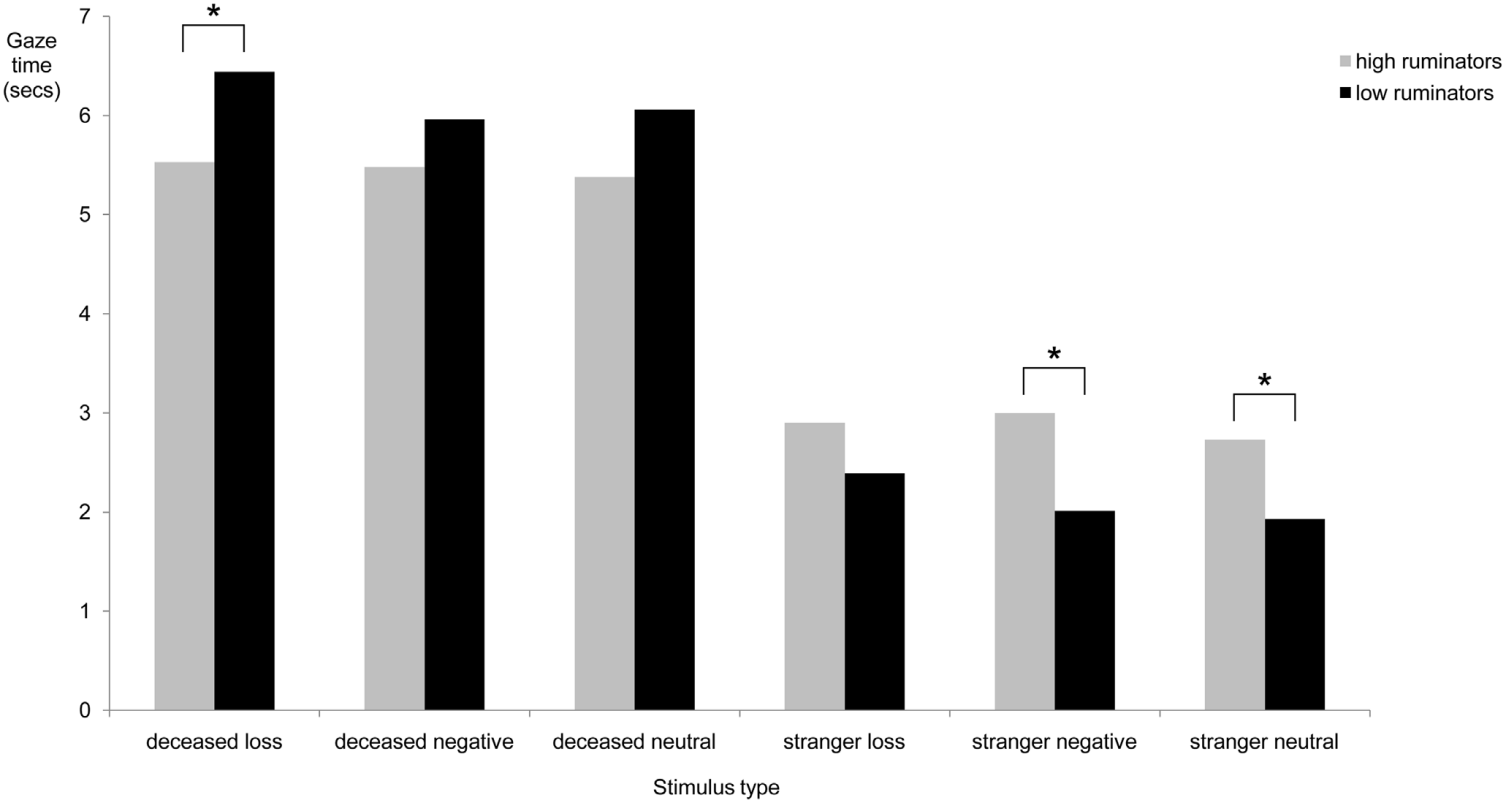
Mean gaze times in seconds for each stimulus type in 1500–10000 ms presentation time. Note. Gaze time is defined as the overall time in seconds spent looking at a picture-word combination (i.e., deceased+loss word, deceased+negative word, deceased+neutral word, stranger+loss word, stranger+negative word, stranger+neutral word) during a specific interval. * = *p*<.05.

**Figure 3:**
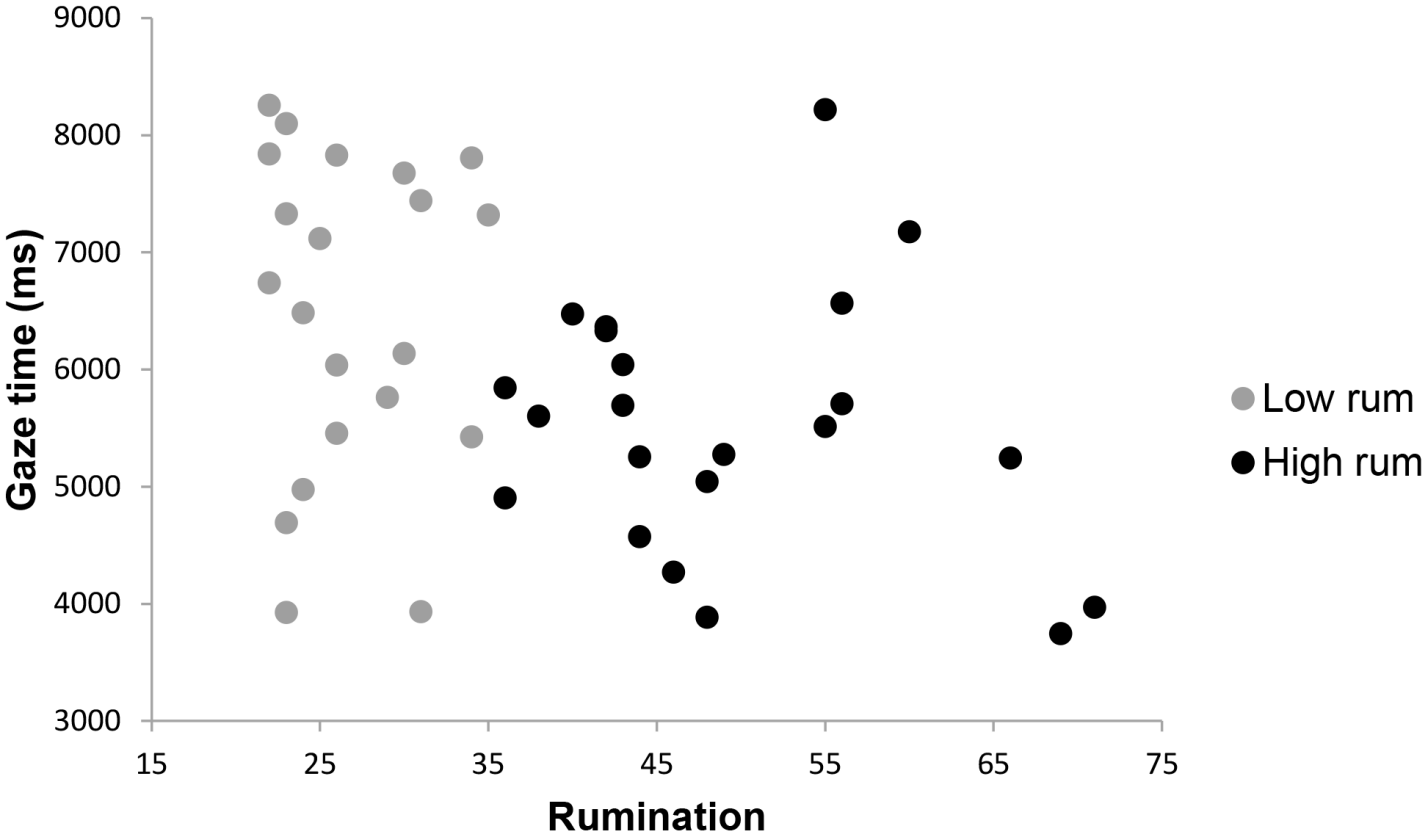
Scatterplot of rumination with mean gaze time in ms in the 1500–10000 ms interval for a picture of the deceased combined with a loss word. Note. Gaze time is defined as the overall time in seconds spent looking at a picture-word combination during a specific interval.

**Figure 4:**
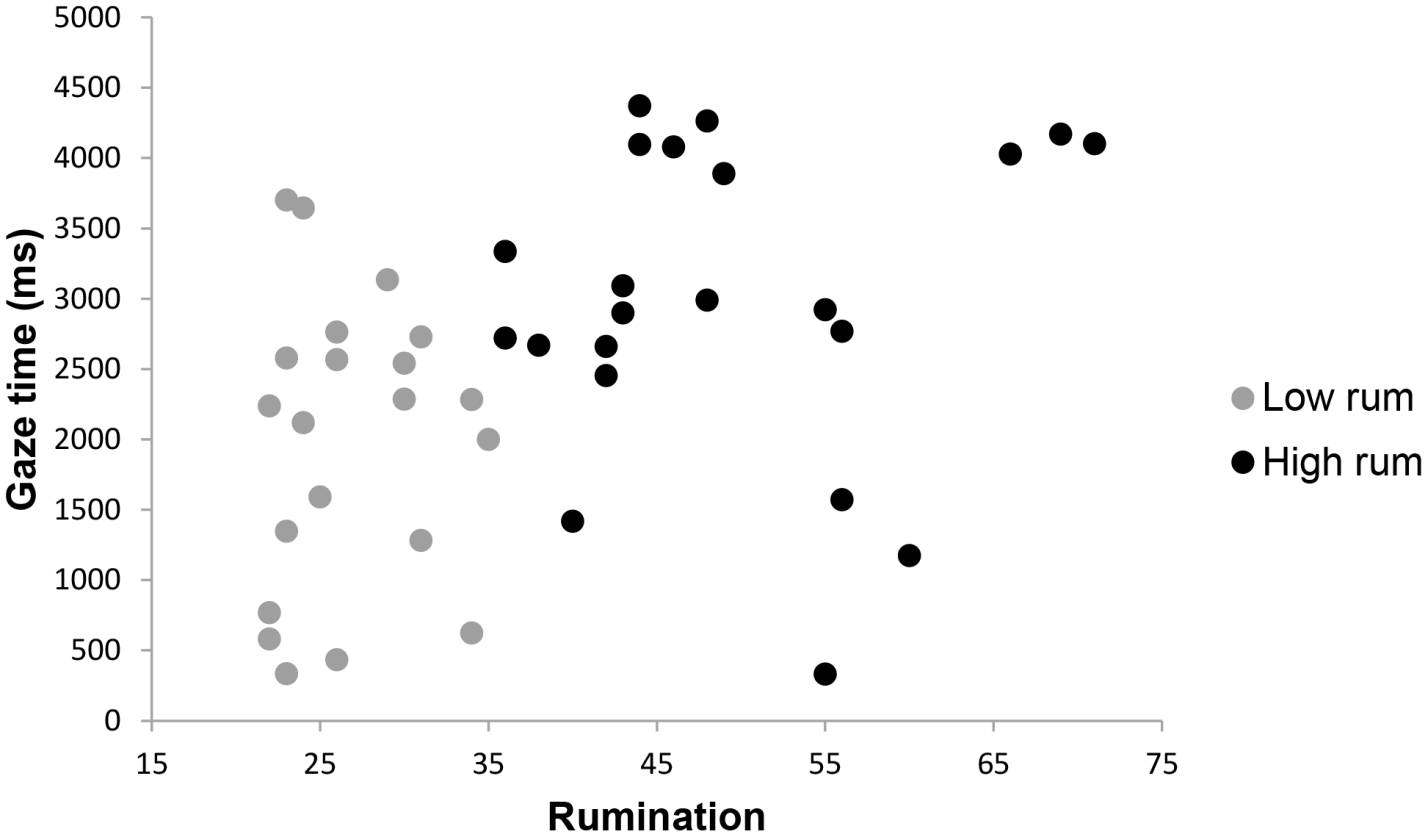
Scatterplot of rumination mean gaze time in ms in the 1500–10000 ms interval for a picture of a stranger combined with a negative word. Note. Gaze time is defined as the overall time in seconds spent looking at a picture-word combination during a specific interval.

To assess long-term attentional bias of high and low ruminators for different stimuli, average gaze times after the initial 1500 ms (1500 ms–10000 ms) were compared for each stimulus type. A 2×6 analysis of variance showed a significant overall effect for rumination, *F* (6,32) = 2.98, *p* = .02, *p*η^2^ = .36, indicating that a difference in gaze times existed between high and low ruminators for one or more stimulus types. Control variables depressive and complicated grief symptoms showed no significant effects on gaze times, *F* (6,32) = 0.40, *p* = .83, and, *F* (6,32) = 1.16, *p = *.35, respectively. Next, hypotheses regarding the differences in gaze times were assessed by comparing high and low ruminators on gaze time for each stimulus type. Conform expectations, high ruminators looked significantly less at pictures of the deceased combined with a loss word than low ruminators, *F*(1,37) = 3.07, *p* = .04, *p*η^2^ = .08. Moreover, compared to low ruminators, high ruminators spent more time looking at pictures of a stranger combined with negative words, *F*(1,37) = 4.92, *p* = .02, *p*η^2^ = .12, and neutral words, *F*(1,36) = 3.67, *p* = .03, *p*η^2^ = .09. No other group differences on gaze time in the 1500–10000 ms interval were found for other stimuli types. These results confirmed the hypothesis that rumination is linked with loss avoidance. Means and standard errors for mean gaze times are shown in Table 2 and are graphically depicted in Figure 2, 3 and 4.

**Table 2:** Mean gaze times and standard deviations in seconds for each stimulus type in 1500–10000 ms presentation time.

Stimulus/Group	Deceased Loss	Deceased Negative	Deceased Neutral	Stranger Loss	Stranger Negative	Stranger Neutral
High ruminators	5.53 (1.10)*	5.49 (1.23)	5.38 (1.25)	2.90 (1.10)	3.00 (1.11)*	2.70 (1.14)*
Low ruminators	6.49 (1.36)*	6.00 (1.47)	5.98 (1.39)	2.36 (1.51)	1.97 (1.02)*	1.93 (1.27)*

Note. Gaze time is defined as overall time spent looking at a stimulus during a specific interval. * = significant difference at p<.05 between high and low ruminators.

#### Analyses of average fixation times

**Figure 5:**
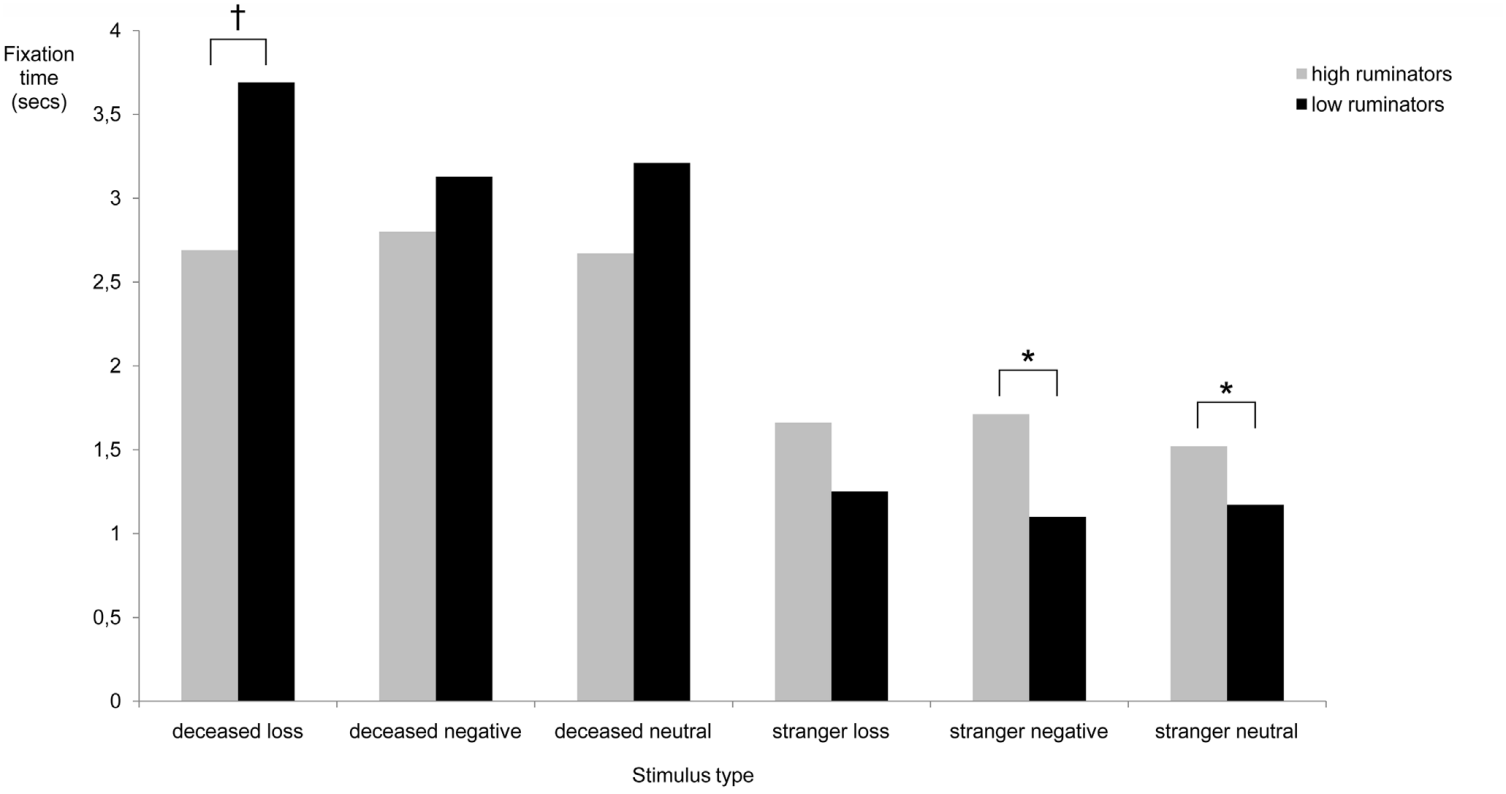
Mean fixation times in seconds for each stimulus type during 0–10000 ms presentation time. Note. Fixation time is defined as the average time in seconds spent looking at a specific picture-word combination (i.e., deceased+loss word, deceased+negative word, deceased+neutral word, stranger+loss word, stranger+negative word, stranger+neutral word) each time a participant looks at it. * = *p*<.05. † = *p*<.10.

**Figure 6:**
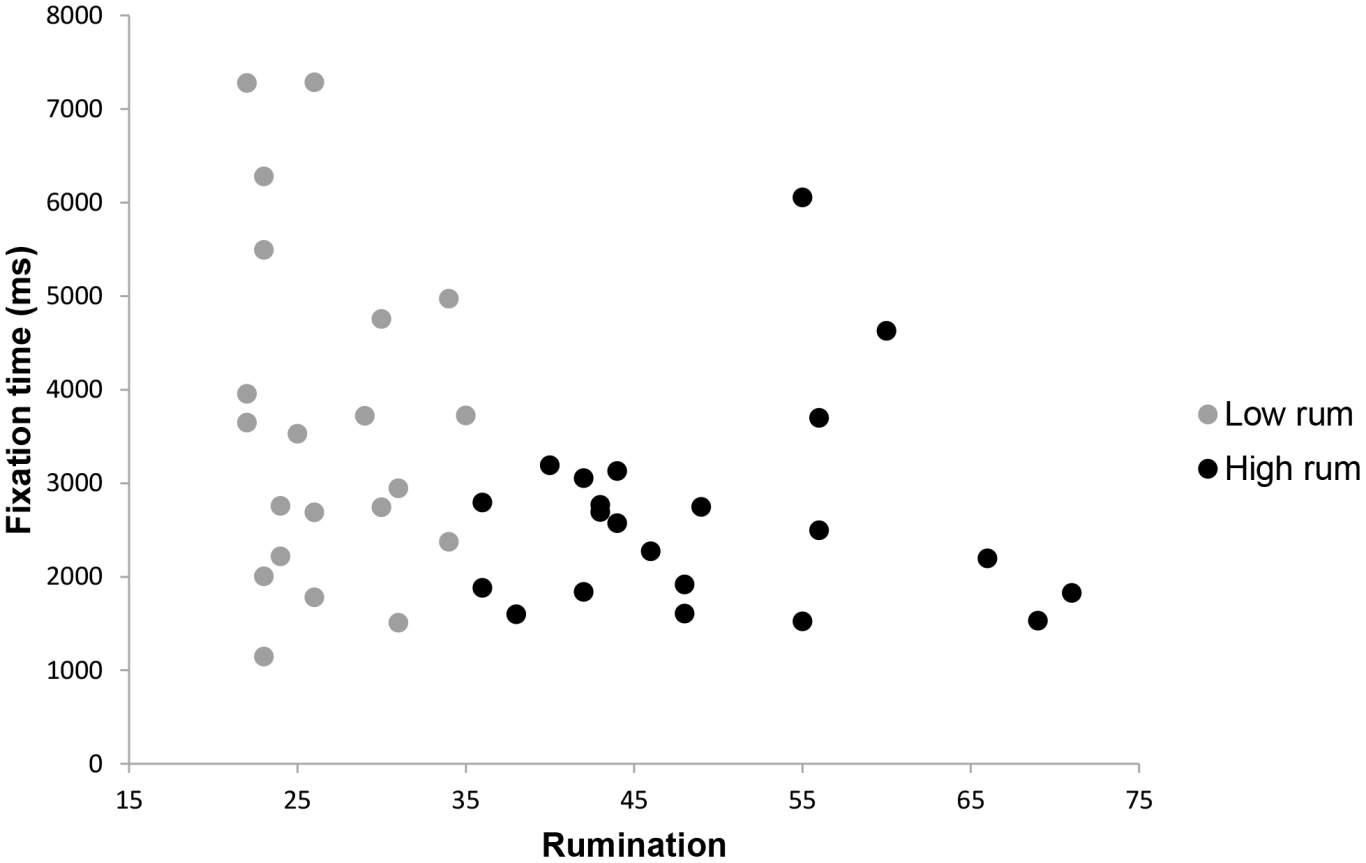
Scatterplot of rumination with fixation time in ms during 0–10000 ms presentation time for a picture of the deceased combined with a loss word. Note. Fixation time is defined as the average time spent looking at a specific stimulus each time one looks at it.

**Figure 7:**
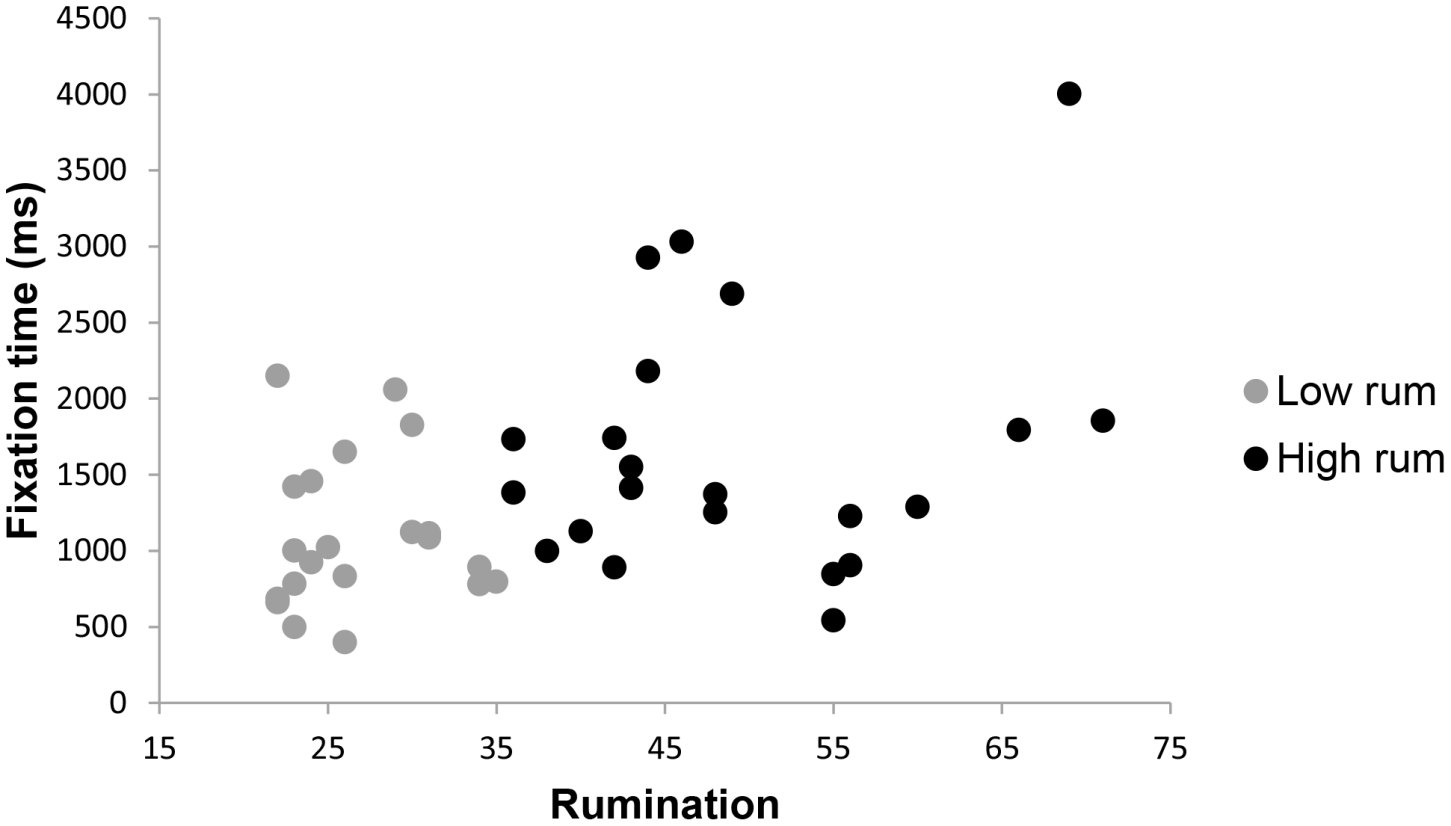
Scatterplot of rumination with fixation time in ms during 0–10000 ms presentation time for a picture of a stranger combined with a negative word. Note. Fixation time is defined as the average time spent looking at a specific stimulus each time one looks at it.

In order to analyse the effects of rumination on average fixation times over the whole 10 second interval a second 2×6 analysis of covariance was conducted. For this outcome variable, the overall test for rumination was marginally significant, *F* (6,32) = 2.16, *p* = .07, *p*η^2^ = .29. The control variables depressive symptoms and complicated grief symptoms showed no significant effects on fixation times, *F* (6,32) = 0.86, *p* = .53. and, *F* (6,32) = 1.47, *p* = .22, respectively. Given the large effect size in the overall test for rumination, and our relatively small sample size, this effect was examined further by comparing high and low ruminators on average fixation times for each stimulus type. High ruminators, compared to low ruminators, showed a trend for shorter fixation times for pictures of the deceased combined with a loss-related word, *F*(1,37) = 2.02, *p* = .08, *p*η^2^ = .05. In contrast, high ruminators showed significantly longer fixation times than low ruminators for pictures of a stranger combined with negative words, *F*(1,37) = 6.43, *p* = .01, *p*η^2^ = .15, or neutral words *F*(1,37) = 4.00, *p* = .03, *p*η^2^ = .10. No other differences between groups were found for fixation times for other stimuli types. These results corroborate findings on gaze times, and provide additional preliminary support for a link between rumination and loss avoidance. Means and standard errors for average fixation times are shown in Table 3 and are graphically depicted in Figure 5, 6 and 7.

**Table 3:** Mean fixation times and standard deviations in seconds for each stimulus type during 0–10000 ms presentation time.

Stimulus/Group	Deceased Loss	Deceased Negative	Deceased Neutral	Stranger Loss	Stranger Negative	Stranger Neutral
High ruminators	2.64 (1.09)^†^	2.75 (1.34)	2.67 (1.29)	1.66 (0.90)	1.67 (0.84)*	1.50 (0.66)*
Low ruminators	3.66 (1.78)^†^	3.10 (1.60)	3.21 (1.79)	1.23 (0.66)	1.10 (0.49)*	1.17 (0.50)*

Note. Fixation time is defined as the average time spent looking at a specific stimulus each time one looks at it. * = significant difference at p<.05 between high ruminators and low ruminators. ^†^ = marginally significant difference at p<.10 between high and low ruminators.

#### Additional analyses

In the analyses described above, the control variables depressive and complicated grief symptoms were added simultaneously to each model. Since symptom levels of depression and complicated grief were highly correlated, *r*(52) = .85, *p*<.001, there may have been content overlap between the two control variables. This suggested that results would be highly similar if we corrected exclusively for one type of distress (depressive symptoms or complicated grief symptoms) in our analyses. To test this idea, we conducted the repeated main analyses, using either depressive symptoms or complicated grief symptoms as a control variable. The effects of rumination on the overall test and post-hoc tests on gaze times (1500–10000 ms) and fixation time (0–10000 ms) were indeed highly similar. Two notable exceptions existed in the models that excluded depressive symptoms as a control variable. First, the overall effect of rumination was significant in the model on fixation time (0–10000 ms), *F*(6, 33) = 2.49, *p* = .04, *p*η^2^ = .31. Second, complicated grief symptom severity was a significant predictor of gaze times (1500–10000 ms), *F*(6, 33) = 2.48, *p* = .04, *p*η^2^ = .31, but yielded no significant post-hoc effects.

## Discussion

The results observed in this study provided no evidence for the hypothesis that high ruminators, compared to low-ruminators, show stronger initial vigilance and subsequent disengagement for loss-reality stimuli. However, high ruminators showed avoidance of stimuli that represent the loss on extended exposure durations (1500 ms–10000 ms). Compared to low ruminators, high ruminators looked less at pictures of the deceased combined with a loss word and more at the picture of a stranger combined with negative or neutral words during this interval. High ruminators also showed a trend to fixate for shorter time periods on pictures of the deceased combined with a loss word than low ruminators. Furthermore, they showed significantly longer average fixation times for pictures of a stranger combined with negative and neutral words than low ruminators. Since analyses were controlled for symptom levels of depression and complicated grief, factors that are associated with attention biases toward negative and loss-related material [45], [46], the current results provide the first evidence for an association between rumination levels and a behavioral measure of loss avoidance (cf. [15], [19]), that cannot be explained by loss-related distress. Effects were mostly medium in size [47], and are in line with results from survey studies reporting significant linear associations between rumination and cognitive and experiential avoidance in bereaved [17], [24] and non-bereaved samples (e.g., [20], [23], [25]–[29]).

Interestingly, findings support the idea that rumination is related to avoidance of personally-relevant threatening material, when less-threatening negative (and neutral) material is simultaneously available. Moreover, no attentional avoidance was found for stimuli that were loss-related, but ambiguous. This supports the hypothesis that rumination may be linked with avoidance of material that unambiguously represents a highly emotional, personally-relevant topic (cf. [23]).

An unexpected finding was that no evidence was found for effects of rumination on attentional biases in the first 1500 ms of exposure time, whereas attentional biases were found for exposure times beyond 1500 ms. Given the late onset of the observed attention biases, we conclude that rumination potentially contributes to strategic, but not automatic attention processes [32]. It seems logical that avoidance linked with cognitive processing comes into play only after a person consciously perceives a threatening stimulus (i.e., after 1000–1500 ms). However, the underlying reason for this null-result may also be methodological. The measurement of attention with eye-tracking for emotional pictorial stimuli has recently been found to show low internal consistency in the first 1500 ms of presentation time [36]. This may have resulted in increased error variance in the measurement of gaze times in the first presentation intervals (i.e., 0–500, 500–1000, 1000–1500), which has possibly limited our power to detect effects in these intervals.

Some additional remarks about the interpretation of our results are warranted. Apart from differing on loss-relatedness, the pictorial stimuli also differed on familiarity, with the picture of the stranger being more novel than the picture of the deceased. One may argue that this could have influenced the results. For example, high ruminators could have experienced concentration problems during the task [48], leading them to take more time to familiarise themselves with the new face presented to them. However, the current pattern of results contradicts a strong bias due to familiarity, as different patterns of attention were found for picture-word combinations, rather than just images. That is, high ruminators, compared to low ruminators, looked less at the picture of the deceased combined with a loss word, but not if this picture was combined with a negative or neutral word. Conversely, high ruminators exhibited increased attention for the picture of a stranger with negative and neutral words, but not for pictures of the stranger with a loss-related word. So, even if familiarity influenced attention, it did not obscure the differential effects of rumination on attention patterns for stimuli types that were predicted beforehand.

Furthermore, although current results support a link between rumination and avoidance after bereavement, it remains to be investigated through which mechanisms rumination and avoidance are linked. Some authors have proposed that rumination is itself an avoidance process [15]–[16], [19], [29], whereas other researchers have argued that rumination has a reciprocal relationship with avoidance [11], [20], [49]. For instance, Nolen-Hoeksema and colleagues [11] suggested that individuals may attempt to escape from rumination through suppression of negative thoughts. Such suppression logically leads to rebound-effects, making negative thoughts more salient, thereby fuelling ruminative thinking [49]. However, recently it has been suggested that rumination could serve as the thought content used to suppress more threatening cognitive material [17]. While the current results seem more in line with the latter hypothesis, additional studies are needed to test such specific ideas. A potentially interesting line of research could focus on investigating whether ruminative thinking can be used as cognitive content to suppress personally relevant, threatening memories, using a variation on methods used in classical suppression research [50].

Finally, the hypothesis that repetitive thinking (e.g., rumination, worry) is a form of avoidance is not specific to the bereavement area, but has also been presented in research on generalised anxiety disorder [21]–[22], post-traumatic stress disorder [16] and depression [23]. Although surveys quite consistently support associations between repetitive thinking and cognitive and emotional avoidance (e.g., [17], ), diverging theories exist regarding what mechanisms underlie an avoidant function of rumination and worry [17], [21]–[22]. Nevertheless, most theorists agree that repetitive thinking may serve to avoid experiencing strong (changes in) negative emotions. The current study uniquely shows that rumination, perhaps to evade aversive emotional experiences, may also be linked with avoidance of reminders of a stressful life-event. This finding may be of particular importance for research on adjustment to trauma. Researchers have long advocated the idea that rumination after a traumatic life-event may be cognitive avoidance because it is focused on why the event occurred and ‘what if’ type questions, rather than on the experience of the trauma as it actually happened. Such avoidance could potentially block integration of the traumatic event with other autobiographical memories, thereby maintaining post-traumatic stress [16]. Yet, this assumption has never formally been tested. One direction for future research could therefore be to establish if trauma-related rumination is associated with avoidance of reminders of the trauma in attention tasks, or in other tasks assessing avoidance tendencies (e.g., [54]).

This study also has a number of limitations. First, the sample primarily consists of conjugally bereaved women. This is common in bereavement research, and may reflect both a stronger tendency of women to share their feelings and the overrepresentation of women in widowhood [55]. Although we currently have no reasons to assume that the mechanisms under investigation are different for men and women, a replication of this study in a group of bereaved men is recommended. Second, the sample consisted of people who decided to participate in this study even after they were informed that they would be shown pictures of the deceased, combined with various words. Although effects in this investigation were moderate in size, stronger effects on attentional avoidance may be expected for bereaved individuals who avoid reminders of the loss more structurally. Third, in this study we compared groups low and high on rumination on their attention patterns, but did not manipulate rumination, by giving each group specific instructions to induce ruminative thinking (e.g., [56]). Therefore, the nature of the relationship between rumination and avoidance after bereavement needs to be investigated further to determine causality.

Despite these limitations, this study adds to understanding of the link between rumination and avoidance in bereavement [19]. It is the first study that has supported an association between rumination and a behavioral measure of loss avoidance in a bereaved sample. If future research corroborates and extends these findings, this could have important clinical implications. Specifically, distraction and behavioral activation have traditionally been advocated as methods to decrease rumination, because these techniques lift mood and give people less time to ruminate [11]. However, if avoidance underlies the effects of rumination, exposure or acceptance-based interventions may (also) be effective in breaking the ruminative cycle in bereavement, because they counter avoidance tendencies. In support of this line of reasoning, both exposure therapy for post-traumatic stress disorder and mindfulness-based cognitive therapy for depression have been found effective in reducing rumination and levels of psychopathology [57]–[58].
